# A new toothless pterosaur from the Early Cretaceous Jehol Biota with comments on the Chaoyangopteridae

**DOI:** 10.1038/s41598-023-48076-7

**Published:** 2023-12-21

**Authors:** Xiaolin Wang, Alexander W. A. Kellner, Shunxing Jiang, He Chen, Fabiana R. Costa, Xin Cheng, Xinjun Zhang, Bruno C. Vila Nova, Diogenes de Almeida Campos, Juliana M. Sayão, Taissa Rodrigues, Renan A. M. Bantim, Antônio A. F. Saraiva, Zhonghe Zhou

**Affiliations:** 1 Key Laboratory of Vertebrate Evolution and Human Origins, Institute of Vertebrate Paleontology and Paleoanthropology, Chinese Academy of Sciences, Beijing, China; 2 University of Chinese Academy of Sciences, Beijing, China; 3 Laboratory of Systematics and Taphonomy of Fossil Vertebrates, Department of Geology and Paleontology, Museu Nacional/UFRJ, Rio de Janeiro, Brazil; 4 School of Ecology, Sun Yat-Sen University, Shenzhen, China; 5 Laboratory of Vertebrate Paleontology and Animal Behavior (LAPC), Center of Natural and Human Sciences, Federal University of ABC, Campus São Bernardo do Campo, São Paulo, Brazil; 6 College of Earth Sciences, Jilin University, Changchun, China; 7 College of Paleontology, Shenyang Normal University, Shenyang, China; 8 Museu de Ciências da Terra, Companhia de Pesquisa de Recursos Minerais, Rio de Janeiro, Brazil; 9 Department of Biology, Federal University of Espírito Santo, Vitória, Espírito Santo Brazil; 10 Museu de Paleontologia Plácido, Cidade Nuvens, Regional University of Cariri, Crato, Ceará Brazil

**Keywords:** Evolution, Palaeontology

## Abstract

The Chaoyangopteridae is a clade of azhdarchoid pterosaurs that stands out in China, particularly in the Jehol Biota, as a Cretaceous group of medium-sized and high-crested pterosaurs. Herein, we describe a new species, *Meilifeilong youhao* gen. et sp. nov., based on two specimens, one tentatively referred to this taxon. This new species represents the most complete and well-preserved chaoyangopterid recorded to date. Along with a set of characters (low premaxillary crest above the nasoantorbital fenestra extending posteriorly, posterior premaxillary process arched and curving posteriorly, a slightly convex sternal articulation surface of coracoid, and a fibular shaft close to proximal articulation strongly arched posteriorly), this species also provides new information both on the unknown palatal region of this clade, and on the rarely preserved (in place) ear portion with stapes. Moreover, *M. youhao* sheds light on paleoecological aspects, while also giving new information about the taxonomic diversity of this peculiar group of Jiufotang pterosaurs.

## Introduction

Pterosaurs comprise an important and enigmatic group of Mesozoic flying reptiles that first evolved active flight among vertebrates, and have filled all aerial environmental niches for almost 160 my^1–3^. Despite being a totally extinct group, they have achieved a wide diversity of forms in a window of time spanning from the Late Triassic to the end of the Cretaceous period^2^. Notwithstanding being found on every continent^2,4^, China stands out by furnishing several new specimens that revealed not only different species, but also entire new clades, such as the azhdarchoid Chaoyangopteridae^5^. This Cretaceous group of medium-sized and high-crested pterosaurs is particularly well known in the Jehol Biota, which includes *Chaoyangopterus zhangi* (formerly considered a nyctosaurid^6^) and *Shenzhoupterus chaoyangensis*^5^ (at the time of description the only preserved posterior region of a skull of a chaoyangopterid, which made clear that those toothless pterosaurs formed a new clade). Other taxa whose phylogenetic position were not clear when first described were subsequently referred to this pterosaur group such as *Jidapterus edentus*^5,7,8^ and *Eoazhdarcho liaoxiensis*^5,9^. The fifth species originally included in the Chaoyangopteridae was *Eopteranodon lii*^5,10^, which has since been regarded as a tapejarid^8,11–13^. More recently, after we have submitted an earlier version of this paper, another species referred to this group was described and referred to *Shenzhoupterus* (*S. sanyainus*^14^).

Here we report a new toothless pterosaur based on two specimens, one of which is the most complete and well-preserved chaoyangopterid recorded to date. The holotype (IVPP V 16059) is particularly well preserved and represents an individual that had a maximized wingspan [maxws^15^] of about 2.16 m. This size is basically the same as *Meilifeilong sanyainus* [maxws 2.18 m] and, along with other features (see Discussion) suggests that they represent distinct species of the same genus. The new taxon also provides several novel information regarding these rather enigmatic flying reptiles, including data on the palatal region. Furthermore, it shows the stapes preserved in place, which is a rare occurrence within pterosaurs.

## Results

### Systematic Paleontology

Pterosauria Kaup, 1834

Pterodactyloidea Plieninger, 1901

Dsungaripteroidea Young, 1964 sensu Kellner 2001, 2003

Azhdarchoidea Nessov 1984, sensu Kellner 2001, 2003

Chaoyangopteridae Lü et al. 2008

*Meilifeilong* gen. nov.

**Type species.** *Meilifeilong youhao*.

**Etymology**. From the Chinese expressions *meili*—beautiful, *fei*—fly, and *long*—dragon, meaning beautiful flying dragon in allusion to the magnificent preservation of the holotype, IVPP V 16059.

**Included species**. *Meilifeilong sanyainus*^14^ comb. nov.

**Diagnosis.** Chaoyangopterid with low premaxillary crest above the nasoantorbital fenestra that extends posteriorly; posterior premaxillary process arched and curving posteriorly; humerus about 20% longer than third phalanx of the wing finger (hu/ph1d4 ~ 1.20).

*Meilifeilong youhao* sp. nov.

**Etymology.** *Youhao*, meaning friendship in mandarin, to celebrate the two-decade continuous collaboration between Chinese and Brazilian paleontologists in pterosaur research since 2003.

**Figure 1:**
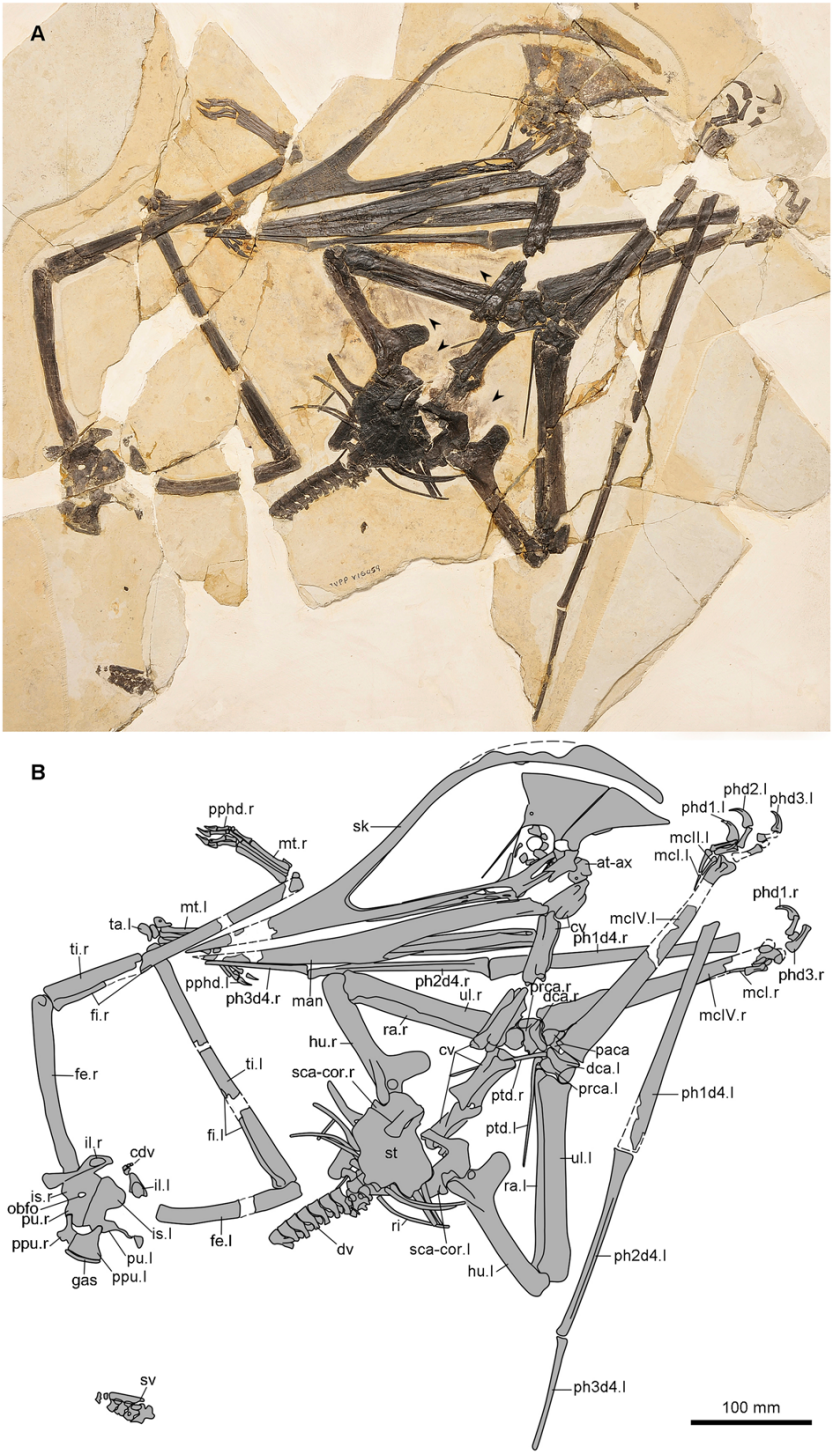
Photo (**A**) and line drawing (**B**) of the holotype (IVPP V 16059) of *Meilifeilong youhao* gen. et sp. nov. Arrows indicate preserved soft tissue. Abbreviations: at-ax, atlas-axis; cdv, caudal vertebra; cv, cervical vertebra; dca, distal syncarpal; dv, dorsal vertebrae; fe, femur; fi, fibula; gas, gastralia; hu, humerus; il, ilium; isc, ischium; man, mandible; mcI-IV, metacarpal I-IV; mt, metatarsal; obfo, obturator foramen.; paca, preaxial carpal; ph1-3d4, first to third phalanx of manual digit IV; phd 1–3, phalanx of the manual digit IIII; pphd, phalanx of the pedal digit; ppu, prepubis; prca, proximal syncarpal; ptd, pteroid; pu, pubis; ra, radius; ri, rib; sca-cor; scapulocoracoid; sk, skull; sv sacral vertebra; st, sternum; ta, tarsal; ti, tibia; ul, ulna; l, left; r, right.

**Figure 2:**
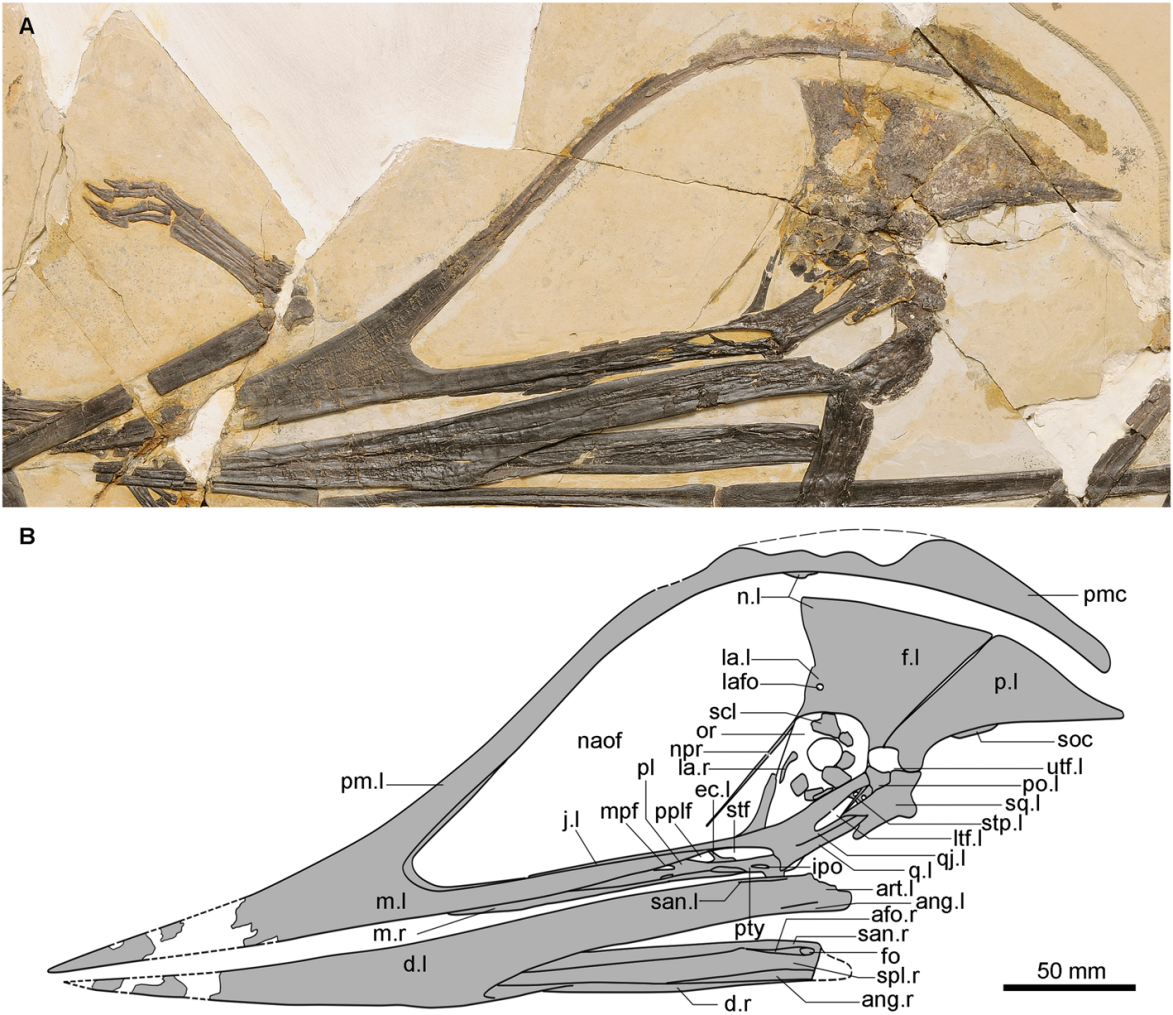
Photo (**A**) and line drawing (**B**) of the cranial elements of the holotype (IVPP V 16059) of *Meilifeilong youhao* gen. et sp. nov. Abbreviations: afo, adductor fossa; ang, angular; art, articular; ch, choana; d, dentary; ec, ectopterygoid; f, frontal; fo, foramen; ios, interorbital septum; ipo, interpterygoid opening; j, jugal; la, lacrimal; lafo, lacrimal foramen; ltf, lower temporal fenestra; n, nasal; naof, nasoantorbital fenestra; npr, nasal process; m, maxilla; mpf, maxillopalatine fenestra; or, orbit; p, parietal; pl, palatine; pm, premaxilla; pmc, premaxillary crest; po, postorbital; pplf, postpalatinal fenestra; pty, pterygoid; ptyf, pterygoid fenestra; q, quadrate; qj, quadratojugal; scl, sclerotic ring; soc, supraoccipital; sq, squamosal; stf, subtemporal fenestra; utf, upper temporal fenestra; vo, vomer; l, left; r, right.

**Figure 3:**
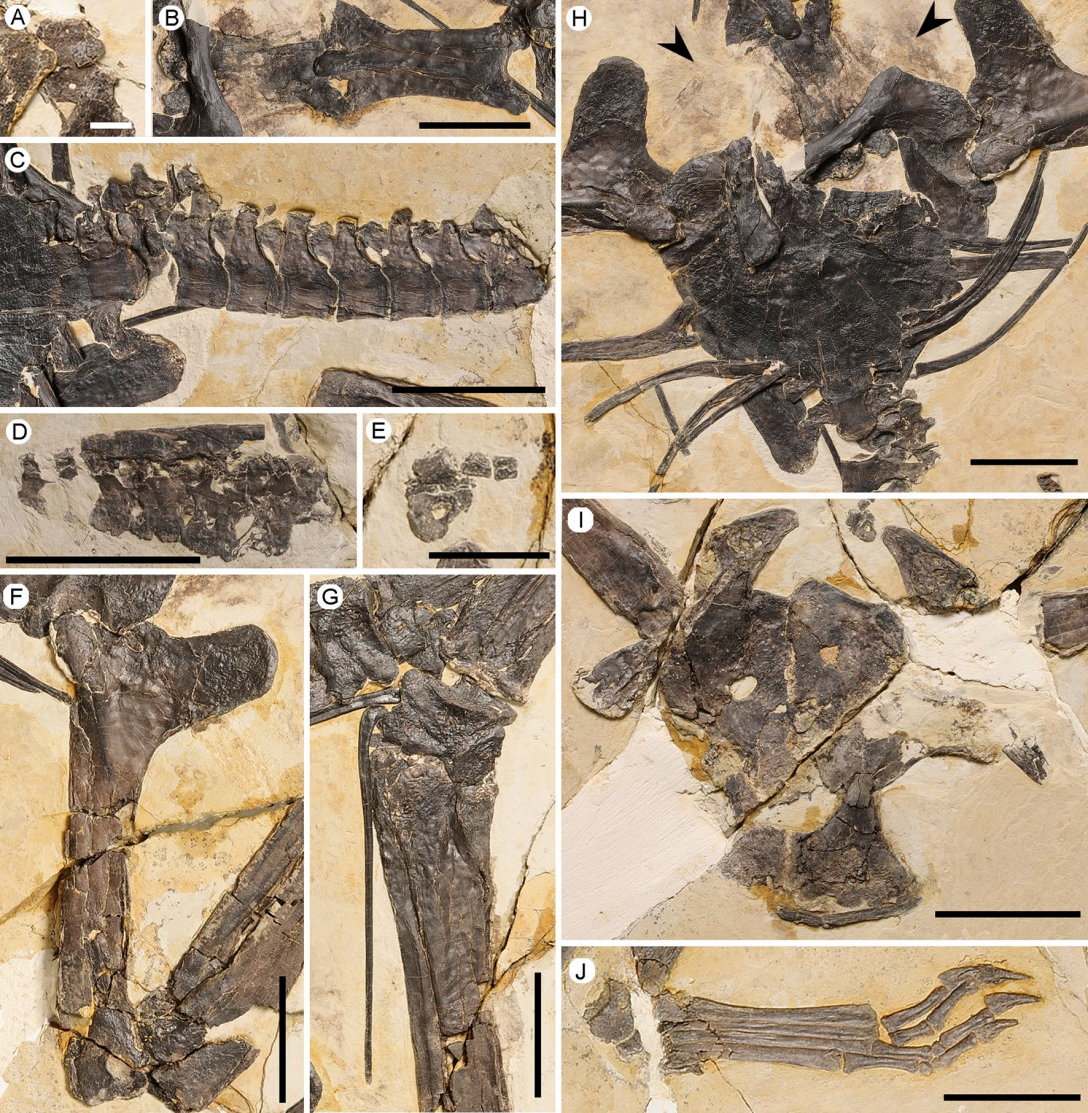
Close-up of the postcranial elements of the holotype (IVPP V 16059) of *Meilifeilong youhao* gen. et sp. nov. (**A**) atlas-axis, (**B**) cervical vertebrae, (**C**) dorsal vertebrae, (**D**) sacral vertebrae; (**E**) caudal vertebrae, (**F**) left humerus, (**G**) left carpal region, (**H**) sternum and pectoral girdles, soft tissue was indicated by arrows; (**I**) pelvic region, (**J**) left foot. Scale bars, 10 mm in A & E, 30 mm in the others.

**Holotype.** IVPP V 16059, an almost complete skeleton housed at the Institute of Vertebrate Paleontology and Paleoanthropology (IVPP), Chinese Academy of Sciences, Beijing, China, Figs. 1, 2 and 3.

**Figure 4:**
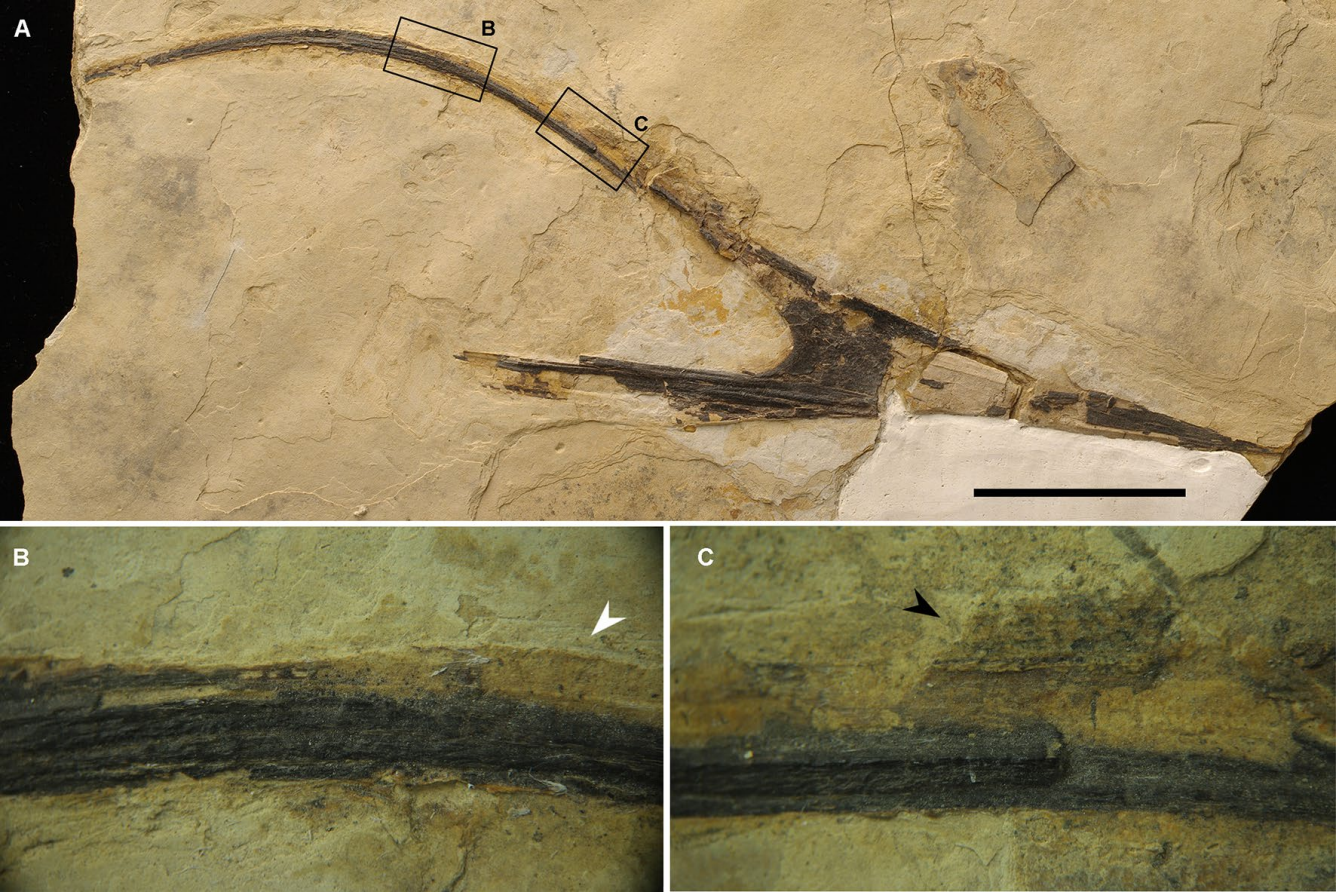
Photos of the referred specimen (IVPP V 17955) of *Meilifeilong youhao* gen. et sp. nov. (**A**) the whole specimen, (**B**) close-up of the bone impression, indicated by a white arrow, (**C**) close-up of the soft tissue, indicated by a black arrow. Scale bar, 50 mm.

**Referred specimen.** IVPP V 17955, partial skull composed of premaxillae and maxillae, Fig. 4.

**Horizon and locality.** Xiaotaizi, Jianchang, Huludao City, western Liaoning China; Jiufotang Formation, Early Cretaceous (Barremian-Aptian).

**Diagnosis.** Chaoyangopterid pterosaur exhibiting the following autapomorphies: sternal articulation surface of coracoid slightly convex; fibular shaft close to proximal articulation strongly arched posteriorly. The new species can be further distinguished from *Meilifeilong sanyainus* by the following features: posterior premaxillary process more arched; jugal with lacrimal and postorbital processes at a narrower angle (~ 45° contra ~ 55° in *Meilifeilong sanyainus*); deeper mandibular symphysis; cervical vertebrae 5 slightly longer than cervical 4; rectangular sternum that is wider than long (square in *Meilifeilong sanyainus*); scapula comparatively longer relative to the coracoid, metatarsal III and IV proportionally shorter than metatarsals I and II.

### Description

The holotype of *Meilifeilong youhao* (IVPP V 16059) is the most complete and well-preserved skeleton of a chaoyangopterid recovered so far (Figs. 1, 2, 3). It consists of essentially all bones, except for most of the tail. The referred specimen (IVPP V 17955) is composed solely by the premaxillae-maxillae and the anterior portion of the palatines and represents a smaller individual (Fig. 4). The description below is based on the holotype unless otherwise noted.

Both specimens are flattened, a common condition of pterosaur remains [e.g.,^16^]. Bones have a dark color that creates a striking contrast with the surrounding whitish cream-colored matrix, enhancing the beauty particularly of the holotype (IVPP V 16059). The bone surface is rather smooth and well preserved except for small taphonomic cracks and breakages. Some portions are missing and were likely lost during the collecting process. Soft tissue is scarce and only found close to the skull and scattered in few areas in the holotype (Figs. 1, 3) and on the dorsal surface of the skull of the referred specimen (Fig. 4).

Regarding taphonomic aspects, nothing special can be noted regarding the referred specimen (IVPP V 17955), which consists of a partially isolated skull. The holotype (IVPP V 16059), however, is rather complete and we assume that the carcass was transported to a body of water, where it sank and reached the bottom intact, not being buried immediately. Due to the likely action of scavengers (e.g., fishes) and currents, the pelvic region was moved away from the main portion of the body and is partially dismembered, what must have happened during the decomposition process. All ribs from the mid-dorsal and posterior vertebrae are missing, suggesting that the carcass was dragged away by currents before final burial. We also noted that the right coracoid, while in almost anatomical position, was broken and the remaining part of the scapulocoracoid pushed against the body of the animal. Such an odd breakage is not commonly observed in pterosaurs and might have occurred before the carcass had reached the bottom where, after some exposure, it was buried. It seems likely that this bone broke while the animal was still alive. Although tempting, it is not known what caused this breakage and whether it contributed to this individual's death.

In the holotype (IVPP V 16059), the skull is exposed in left lateral view, with part of the palatal region ventrally displaced relative to its lateral margin (Fig. 2). The nasoantorbital fenestra is the largest opening in the skull, with tall posterior margin and dorsal margin positioned just above the dorsal margin of its pear-shaped orbit. The skull also bears a rounded upper temporal fenestra and an elongated, almost slit-like lower temporal fenestra. Inside, a thin rod-like element—the stapes—can be seen, which is rarely preserved in pterosaurs.

Regarding the palate (here we follow the new terminology of Chen, 2021^17^), the largest opening is the choana (minimum length—80 mm) while the elongated maxillopalatine fenestra is the smallest. The postpalatine fenestra is oval, and the shape of the subtemporal fenestra is an elongated ellipse. There is also a small, longer than wide interpterygoid opening.

The rostrum is long, slightly inclined ventrally relative to the larger margins of the skull (~ 5°) and with a Rostral Value (RV, sensu Kellner^16^) of 7.89. The premaxilla is the largest cranial element, with its external surface grooved by longitudinal lines and without a discernible suture with the maxilla. Anteriorly, this bone bears a triangular cross-section and tappers gradually ending in a pointed tip. It also has a very long and arched dorsal process that extends posteriorly beyond the occipital region and runs above the frontals and the parietals without contacting them. The cleft resulting from this lack of contact could have been either filled with soft tissue or (more probably) was caused by the dorsal displacement of the premaxillae. The dorsal surface of the posterior premaxillary process is partially broken, but a low bony premaxillary sagittal crest at the mid-part of the nasoantorbital fenestra that gets gradually taller towards the posterior region can be seen. The external surface of this crest is rugose in some areas, particularly at the posterior region, which could suggest this structure being roofed by a horny covering or soft tissue. No branching structures as reported in some other pterosaurs^15,18^ were observed.

As the premaxillae, a thickened bone with rounded edges forms the ventral lateral border of the maxilla. The palatal surface changes from flat to slightly concave towards the posterior region. There is a rugose surface between the dorsal and ventral margins at the premaxilla-maxilla just anterior to the nasoantorbital fenestra that shows some vertical bony struts due to compression. These elements are likely from the internal trabecular system and can suggest a pneumatic anterior portion of the rostrum. An also rugose, thickened bony rim is present at the anterior margin of the nasoantorbital fenestra.

The nasal is placed at the posterior end of the nasoantorbital fenestra, just above the lacrimal and in a quite unusual position, with an almost vertical main body. No clear sutures with the surrounding elements are discernible. A small element at the ventral portion of the posterior dorsal process of the premaxillae is likely part of the nasal. The nasal process is a very long, delicate, anteroventrally directed and tappers to a pointed end almost reaching the ventral border of the nasoantorbital fenestra. No foramen piercing this element was observed.

The skull roof comprises fused frontals and parietals that are overlain (but not contacted) by the posterior dorsal premaxillary process. Its surface shows several cracks through which internal imprints of a developed trabecular system can be seen. There is no clear frontal crest, but the frontal forms the basis of the premaxillary crest. Notwithstanding, the parietal must have formed a crest that had likely extended backwards above the occipital region. There are several cracks through which internal imprints of a developed trabecular system is observed. This provides evidence of a pneumatic system above the braincase as has been observed in other pterodactyloids^19^.

The lacrimal is not well preserved and bears a thin ventral process that overlies the jugal to form with the latter a bony bar separating the nasoantorbital fenestra from the orbit. It has an extra, ventrally oriented short process lying parallel to the jugal process that ends in a sharp point. Although some small openings at the lateral side of the lacrimal are observed, this element does not seem to be fenestrated as reported in some tapejarids (e.g.,^20^). The postorbital is not well preserved. It is a flattened bone that forms both the lateral margin and the dorsal border of the upper and lower temporal fenestrae, respectively.

The jugal has three elongated processes: a broad postorbital process, a thin and elongated maxillary process placed just above the maxilla that does not reach the anterior margin of the nasoantorbital fenestra (as in *Chaoyangopterus*), and a thin lacrimal process that is inclined posteriorly (~ 115°) (similar to *Shenzhoupterus chaoyangensis*^5^).

The limits of the bones forming the posterior region of the skull (e.g., the quadrate, quadratojugal, and squamosal) are hard to establish due to fusion. The quadratojugal forms the ventral and posterior margins of the lower temporal fenestra, at least partially excluding the quadrate from this opening. This latter is an elongated and posteriorly inclined (~ 160°) element. The articulation surface for the mandible is formed by large lateral and medial condyles that are separated by a wide saddle-shaped concave surface. The cranio-mandibular articulation is positioned under the anterior half of the orbit. The squamosal has a comparatively broad and short otic process that ends in a rather blunt surface and is probably incomplete.

The stapes is preserved in what seems to be its correct anatomical position (i.e., inside the lower temporal opening). It is a delicate rod-like element that tapers ventrally. As both of its ends are partially covered by bones and matrix, only its minimum length (14.5 mm) can be presented.

Some palatal bones are observed since they have been ventrally displaced. The maxillopalatine fenestra is formed by the maxilla and the anterior and lateral processes of the palatine. An elongated, rod-like bone partially exposed in this fenestra is tentatively regarded as the vomer. The pterygoid is fused with the posterior part of the palatine. The base of the ectopterygoid mostly covered by the pterygoid and its lateral process is thin, which separates the postpalatinal and the subtemporal fenestrae. The maxilla-palatine bar forms the lateral margin of the choana.

Not many details of the occipital region are observed. The sole element that could be identified is the supraoccipital that extends posteriorly and forms the base of the premaxillary crest.

The referred specimen (IVPP V 17955) is composed of the premaxillae-maxillae, exposed in right lateral view. Part of the lower margins at the left side was displaced ventrally during the fossilization process (Fig. 4). Despite representing a ~ 17% smaller individual than the holotype (based on the rostrum length), it shows a quite similar morphology to the holotype: some rostral shape and arched posterior process of the premaxilla. It further shows a low sagittal bone structure here regarded as part of the premaxillary crest as observed in the holotype. The rostrum is elongated and presents a slightly lower RV (7.60). It also shows a thickened rugose bony rim forming the anterior margin of the nasoantorbital fenestra, which is more developed in this specimen than in the holotype. Regarding the palatal elements, only the anterior portion of the palatines are partially exposed and border the anterior margin of the choana.

The mandible is slightly displaced posteriorly with respect to the skull and exposed in left lateroventral view, with its right ramus ventrally displaced and displaying its medial portion. This bone bears a single, dorsoventrally compressed opening—the adductor fossa (length—19.4 mm). The dentary, typically the largest bone of the lower jaw in pterosaurs, forms a moderately deep, long symphysis that tapers anteriorly to a pointed tip as the rostral end. A dentary fossa (sensu Kellner^21^) from the two fused segments of the dentary is about 15.9 mm long. This bone bears a rounded ventral margin and no dentary crest, and has apparently reached the mandible's posterior end. The dentary laterally overlays the angular, which appears to be fused with the former while medially extending as a split-like element that tapers between the splenial (a smooth, thin element at the medial portion of the mandibular rami) and the dentary. The angular forms the posterior part of the articulation surface that receives the quadrate, and the surangular the dorsal margin of the posterior region of the lower jaw. The latter contributes to the adductor fossa at its dorsal margin and lateral wall (Fig. 2). There is no evidence of a prearticular.

Several bony plates form the sclerotic ring. There are more than 10 elements with diameters about 11.5 mm.

The first seven vertebrae from the cervical series are exposed either laterally or ventrally. The atlas-axis complex, not well preserved, seems to be fused. The axis bears a tall neural spine and is laterally perforated by a pneumatic foramen (Fig. 3A). Cervical vertebrae 3–7 are elongated and bear similar morphological features. All bear low blade-like spines and, where observable, show developed hypapophyses (Fig. 3B). The centrum is not pierced by a pneumatic foramen as observed in several pterodactyloid pterosaurs (e.g.,^22–24^). Cervical vertebra 5 (57.2 mm) is slightly larger than cervical 4 (56.8 mm), followed by cervicals 6 (52.0 mm) and 7 (42.6 mm). This differs from the condition observed in *Meilifeilong sanyainus*, where cervical 4 is larger than cervical 5^14^.

From cervical 7 all remaining vertebrae, including the anterior dorsals, are covered by the sternum. A total of 10 dorsal vertebrae are observed exposed in ventral or left ventrolateral view (Fig. 3C). All are procoelous with the first two showing fused centra, indicating the presence of a notarium. The second observed dorsal shows a well-developed foramen at the ventral surface of the diapophyses, bearing like all others, a low neural spine. No pneumatic foramina were observed on the lateral surface of the centra.

The sacrum is formed by six elements exposed in right dorsolateral side (Fig. 3D). An anteroposteriorly elongated supraneural plate formed by the fusion of neural spines of sacrals 2–4 can be identified. It seems that all six sacral vertebrae might likely have taken part in this structure, which cannot be established with certainty since the neural spines of sacrals 1, 5 and 6 are broken. At least four caudals that gradually become smaller towards the posterior region can be observed (Fig. 3E).

Only the ribs of cervicals 8 or 9 and some of the anterior dorsals are preserved. They are double-headed elements, with a more developed capitulum than the tuberculum, and show a pneumatic foramen. The most posterior dorsals lack any ribs that might have been removed during the fossilization process.

The sternum is observable from its ventral side. The cristospine and most of its margin were broken (Fig. 3H). Nevertheless, this bone has a rectangular shape, being wider than long (estimated dimensions: 7.8 × 6.9 mm), with a rather straight lateral margin. The scapula is larger than the coracoid and both bones are fused. The former is an elongated element that bears a broad tuberculum at the posterolateral margin close to a large glenoid fossa. Its proximal articulation is dorsoventrally flattened and convex. The coracoid lacks a ventral flange or a tuberculum, showing a dorsoventrally flattened and slightly convex articulation with the sternum. The right coracoid was broken from the main part of the scapulocoracoid and pushed against the body, below the ventral surface of the sternum.

Nearly complete wings (lacking the fourth wing phalanges) are mostly exposed in ventral view. The long humerus bears a large pneumatic foramen at the base of the deltopectoral crest, close to the humeral head (Fig. 3F). This crest is a large and rectangular structure with a rounded distal end whose sides show a more rugose and thickened bone surface. Radius and ulna are elongated elements, with diameter of the radius about half that of the ulna. The latter has a well-developed *fovea carpalis*, and an unusually rugose portion of the surface at its proximal articulation suggests a pathology. Both proximal and distal carpal series are fused and perforated by several foramina, particularly the distal series (Fig. 3G). The preaxial carpals are pierced by a foramen and show a developed sesamoid. The long pteroid extends to more than half of the ulna length and tapers to a blunt point. At its articulation with the proximal syncarpal (as showed at the left side) this bone is expanded and curved, without any sign of a posterior foramen. Metacarpals I-III are very thin, with expanded distal articulations and shafts tapering proximally. Although metacarpals II and III are incomplete, they unlikely have reached the carpal series. Metacarpal I is more developed and might have reached the carpus. The distal articulation of the left metacarpal IV is pierced by a foramen at the posterior surface. Manual digits I-III are partially preserved and bear curved unguals that are much larger than the pedal ones, and show a large lateral groove. No horny covering could be observed. The first phalanges of manual digit IV lack their proximal articulation and were positioned in close connection with metacarpal IV. No evidence of a ventral ridge or a developed posterior concavity could be observed.

The pelvic girdle is crushed dorsoventrally, and all preserved elements seem to be fused, but not to the corresponding counterpart (Fig. 3I). The ilium was pushed ventrally against the remaining pelvic bones and still bears both postacetabular and parts of the preacetabular processes. A developed postacetabular expansion is separated from the acetabular portion by a short, constricted shaft, with a triangular distal end in lateral view that narrows to a blunt point. The dorsal margin is deflected from this expansion at about 23°. A developed medial postacetabular fossa, filled with trabecular bone, can be observed. There are four to five articular facets for the sacral vertebrae, showing that the sacrum was not fused with the pelvic elements as has been observed in several pterodactyloids (e.g.,^25,26^). An ischiopubic plate is formed and bears a small, oval-shaped obturator foramen whose longer axis is posteroventrally oriented. Anterior to this foramen there is a smaller one, close to the plate's ventral margin. The anterior margin of the pubis is concave. Both prepubes are ventrally exposed and strongly connected, with an apparent suture. These elements are triradiate with short processes that connects them to the pubis and show straight anteroventral margins and expanded ventral portions. Their contact surfaces are relatively long (14 mm) and straight.

The holotype (IVPP V 16059) shows both femora that are overlapped by the pelvis. They present a rather slightly curved shaft. A small protuberance just above the lateral condyle regarded as the lateral epicondyle is observed on the right femur. The presence of a posterior foramen between the femoral neck and the greater trochanter cannot be confirmed. The fibula has an expanded proximal articulation and fuses with the tibia. The right fibula separates from the tibia right below the proximal articulation and arches posteriorly, tapering towards the distal portion until reaching the lateroposterior surface where it fuses with this bone. Although the exact distal limits between these bones are not clear, the fibula is estimated to have reached about half of the tibia´s length. The tarsi from both sides are preserved and are not fused to the tibia, forming a tibiotarsus. The articulation surfaces of the astragalus to the tibia is concave and rounded to the distal tarsals, with both lateral and medial condyles projected. Laterally this bone is pierced by a foramen. Two distal tarsals can be observed, with the medial tarsal being the larger element.

The metatarsals lie parallel to each other, with metatarsal I and II subequal in length, followed by metatarsals III  and IV (Fig. 3J). This configuration differs slightly from *Meilifeilong sanyainus*, in which the size difference between metatarsal III to metatarsals I and II is smaller^14^. In dorsal view metatarsal I is wider than metatarsals II-III. The left metatarsal V bears an oval depression that likely leads to a foramen. The phalangeal formula of the foot is the typical 2:3:4:5:1 configuration of pterodactyloids^26^, with the last digit bearing a single tiny phalanx. The pedal unguals are long but much smaller than the manual ones.

Although the discussion about ontogeny in Pterosauria is still in its infancy^27–29^, the fusion of bones has been regarded as a proxy for ontogenetic development in this (and other) group of vertebrates^27,28^. The following bones are fused in the holotype of *Meilifeilong youhao*: scapula and coracoid, distal and proximal syncarpals, sacral vertebrae (at least the neural spines of sacral vertebrae 2–4), ischium and pubis forming an ischiopubic plate. Sacral vertebrae are not fused to the ischium and it is not clear if the latter is fused with the ischiopubic plate, a feature observed in at least derived pterodactyloids [e.g.,^25^]. Although there is still controversy in how to assess the ontogenetic stages based on fusion of bones within Pterosauria, the degree of fusion of the skeleton represented by the holotype of *Meilifeilong youhao* suggest that it had reached at least an ontogenetic stage 4 (OS4) sensu Kellner 2015^28^, perhaps even OS5.

## Discussion

The overall morphology of *Meilifeilong youhao* (e.g., the edentulous condition, pear-shaped orbit) shows that it belongs within the Azhdarchoidea^22,24^. As diagnostic for this clade, which also includes the Chaoyangopteridae^5,30^, the nasoantorbital fenestra is extremely large compared to other cranial opening, what is also observed in *Meilifeilong youhao*. Its anterior margin is proportionally similar in size to that of *Shenzhoupterus chaoyangensis* and comparatively larger than those of *Jidapterus* and *Chaoyangopterus*. The posterior upper corner of this fenestra extends over part of the orbit, a feature shared with *Shenzhoupterus chaoyangensis*^5^ and *Meilifeilong sanyainus*^14^ and is here regarded as a chaoyangopterid synapomorphy. However, despite its similarities with other chaoyangopterids, *Meilifeilong youhao* can be separated by some autapomorphies and a set of unique combination of characters.

Regarding the palatal region, *Meilifeilong youhao* shows a maxillopalatine fenestra (traditionally called the postpalatine fenestra [e.g.,^21,25^]) with the anterior margin more constricted than the posterior one. This palatal opening does also not overlap laterally the postpalatinal fenestra (secondary subtemporal fenestra^21^) as in the tapejarid *Caupedactylus*. There is also a small, longer-than-wide interpterygoid opening that is different from the rounded condition observed in *Pteranodon*^25^ and the wider-than-long one reported in *Caupedactylus.* Moreover, the rarely preserved stapes can be seen through the lower temporal fenestra of *M. youhao.* Rod-like elements tentatively identified as stapes have been previously seen in a single specimen of *Pteranodon*^25^, but these were longer (21 mm minimum length) than those of the new Chinese species.

Chaoyangopterids bear longer rostra compared to the length of the skull (squamosal-premaxilla) than those of tapejarids. The elongated rostrum of *M. youhao* is proportionally longer than that of *Shenzhoupterus chaoyangensis*, but shorter than those of *Chaoyangopterus* and *Jidapterus*^5–7^, and about the same size of *Meilifeilong sanyainus*. It is slightly deflected ventrally relative to the larger margins of the skull (~ 5°), a different condition observed in tapejarine tapejarids (e.g.,^6,31^). The rostral value^16^ (RV) is 7.89, lower than in *Chaoyangopterus* and *Jidapterus* (10.72 and 19.15, respectively; see Table 1), and similar to the estimated value for *Meilifeilong sanyainus* (8.57). The dorsal posterior process of the premaxilla is arched and longer than in any other chaoyangopterid, which differs from the straight condition observed in *Jidapterus*. This process is also arched in *Shenzhoupterus chaoyangensis* and in *Meilifeilong sanyainus*, but not to the same degree as in the new species.

**Table 1:** Cranial measurements (in mm) of the holotype (IVPP V 16059) of *Meilifeilong youhao*.

Elements	Values
Skull length (from the anterior tip of the premaxilla to the posterior end of squamosal)	345
Rostral length (ros)	142.1
Maximum length of nasoantorbital fenestra	176
Ventral projection of nasoantorbital fenestra	125
Height of the anteromost point of the external naris or nasoantorbital fenestra (aen-h)	18
Rostral Value (ros/aen-h)	7.89
Ros/skull-length	0.41
Choana length	~ 56 (minimum)
Mandible length	302
Mandibular symphysis	~ 177.3
Mandibular symphysis/mandible length	~ 0.59

It is interesting to note the presence of a cleft between the posterior process of the premaxilla that does not connect with the frontal and parietal in both *Meilifeilong sanyainus* and *Meilifeilong youhao*, which has been also observed in one specimen of a tapejarine tapejarid^26^. Although we cannot a priori rule out a potential systematic signal of this feature, it could be the result of a combination between taphonomy and ontogeny, in which the posterior process of the premaxilla was displaced from its natural position above the skull roof during the fossilization process and that in ontogenetically more developed individuals it would eventually fuse with the frontal and parietal.

Based on the fusion of bones, there is a marked difference regarding the ontogenetic stage^28^ of the holotypes of *Meilifeilong youhao* and *Meilifeilong sanyainus*. The individual that is represented by the holotype (IVPP V 16059) of *Meilifeilong youhao* has reached at least OS4. The sole known individual of *Meilifeilong sanyainus*, albeit being almost the same size, shows many more unfused elements (e.g., carpals, sacral vertebrae, scapula and coracoid), suggesting that it was between OS2 and OS3^28^ (in any case, less ontogenetically mature than IVPP V 16059). This could indicate that the posterior premaxillary process might fuse very late in ontogeny.

Another noteworthy mention is the presence of an ossified premaxillary crest above the premaxillary process of *Meilifeilong youhao*. Although the discussion of the function of crest is still an ongoing debate [e.g.,^16,18,25,32–35^) there is some indication that crested pterosaurs show the presence of cranial crests even as hatchlings^32^. In *Meilifeilong youhao* the premaxillary crest is more developed in the holotype than in the referred specimen (Figs. 2, 3), which is smaller and regarded as belonging to a younger animal. This suggests that younger individuals would have had smaller crests that might grow during ontogeny, as has been reported in some other pterosaurs^32^. If it could also be related to sexual dimorphism as observed in some other pterodactyloids^36^, cannot be established in the limited number of specimens of this taxon.

Based on the published figures of the sole specimen known from *Meilifeilong sanyainus*^14^, it also bears a cranial sagittal crest that is lower than in *Meilifeilong youhao*, which is one of the characters that unite these two species.

Where observable, chaoyangopterids have an elongated dentary symphysis that reaches about half the mandibular length (e.g., *Chaoyangopterus*, *Jidapterus*, *Shenzhoupterus chaoyangensis*, and *Meilifeilong sanyainus*). In *Meilifeilong youhao* this symphysis is proportionally longer reaching almost 60% of the length of the mandible. Moreover, the dorsal segment of dentary is more posteriorly located like in the azhdarchid *Quetzalcoatlus*^37^ and in tapejarids^21^.

Where observable, cervical vertebrae lack a lateral pneumatic foramen on the centrum, a different condition from tapejarids^38^. Cervicals 3–7 are elongated, but not such as reported for the Azhdarchidae^39^. According to the original description of *Meilifeilong sanyainus*^14^, cervical 4 is the longest element in the neck of this pterosaur, with cervical 7 being considerably smaller (only 65% of cervical 4^14^). In *Meilifeilong youhao*, cervical 5 is slightly longer than cervical 4 and the proportions in length of the remaining cervical elements are not so disparate as reported in *Meilifeilong sanyainus*^14^.

The sternum of *Meilifeilong youhao* is essentially a rectangular plate, which is wider than long, differing from the semicircular or square sternum reported in Tapejaridae^32,40,41^. This bone also differs that of from the chaoyangopterid *Jidapterus* by having the anterior margin more posteriorly directed, a condition similar to *Chaoyangopterus*^6,7^. The sternum of the new species also differs from the one of *Meilifeilong sanyainus*, which is square^14^.

Scapula-coracoid ratio (sca/cor) is 1.25 (Table 2), with scapula longer than the coracoid. This is similar as in other chaoyangopterids except for *Shenzhoupterus chaoyangensis*, in which the bones have similar lengths^5^. The coracoid lacks both a ventral flange as observed in azhdarchids^37^ and a tuberculum that is found in tapejarids^31^. Its dorsoventrally flattened and slightly convex articulation with the sternum is unlike the condition observed in tapejarids and *Quetzalcoatlus lawsoni*^42^.

**Table 2:** Postcranial measurements (in mm) of the holotype (IVPP V 16059) of *Meilifeilong youhao.*

Elements	Right	Left
Scapula	75.6	–
Coracoid	~ 62.4	–
Humerus	115.5	118.8
Ulna	158.6	162.0
Metacarpal IV	–	205.2
Pteroid	–	92.1
First wing phalange	230^a^	–
Second wing phalange	150.5	160.2
Third wing phalange	~ 95.0	–
Femur	~ 155	–
Tibia	228.0	228.0
Metatarsal III	43.9	–
Metatarsal IV	38.9	–

^a^Estimated value.

The humerus reaches about 60% the length of the wing metacarpal (hu/mcIV = 0.58), differing from *Chaoyangopterus* (hu/mcIV = 0.50^6^) and *Eoazdarcho* (hu/mcIV = 0.67^10^). Based on the published information of *Eoazhdarcho liaoxiensis*^10^, this species differs from all other chaoyangopterids, including *Meilifeilong youhao* by having the first phalanx of the wing finger proportionally smaller than other elements of the wing.

There is a trend within the azhdarchoids to increase metacarpal IV in respect to the humerus, which is also observed in chaoyangopterids, including the new specimen described (IVPP V 16059) here. In tapejarids, the ratio hu/mcIV is 0.60–0.76, while in most chaoyangopterids it is 0.52–0.58, and about 0.40 in azhdarchids (e.g., *Quetzalcoatlus lawsoni*, *Zhejiangopterus linhaiensis*).

Another trend showing enlargement of the metacarpal IV within azhdarchoids can be observed regarding the first wing phalanx (ph1d4/mcIV). Overall, members of the Tapejaridae, Chaoyangopteridae, and Azhdarchidae have a longer metacarpal IV compared to the first phalanx of the wing finger than other pterodactyloids. In tapejarine tapejarids the ratio ph1d4/mcIV tends to be between 1.25 and 1.33^13,40,41,43^ and in chaoyangopterids around 1.15 (including *Meilifeilong youhao*). In azhdarchids, the metacarpal IV is slightly larger than the first phalanx of the wing digit (e.g., *Quetzalcoatlus*^37,42^, *Zhejiangopterus linhaiensis*^44^). This indicates a length increase of metacarpal IV relative to the first wing phalanx in Tapejaridae, Chaoyangopteridae, and Azhdarchidae.

*Meilifeilong youhao* is a new genus and species of a chaoyangopterid pterosaur from the Jiufotang Formation, China, and the best preserved Chaoyangopteridae discovered so far. This new species bears a suite of characters such as low premaxillary crest above the nasoantorbital fenestra that extends posteriorly, posterior premaxillary process arched and curving posteriorly shared with a recently described chaoyangopterid^14^ that is here referred to the same genus—*Meilifeilong sanyainus.* It can further be separated from the latter by several features, including by different proportions of wing elements, a deeper mandibular symphysis, the posterior premaxillary process more arched and the proportions of metatarsals.

Regarding the distribution of Chaoyangopteridae, as has been pointed out before, the vast majority of specimens collected so far come from the Jiufotang Formation that crops out in China. Other reports came from Brazil^45,46^ and from Morrocco^47^.

The first supposed chaoyangopterid occurrence out of China comes from the Lower Cretaceous (Aptian) Crato Formation^45^, a deposit that has yielded several important pterosaur specimens, particularly tapejarids^1,21,28^. The material consists of an incomplete skull very hard to reconstruct that has cast doubts about its referral to the Chaoyangopteridae^16^. However, if the reconstruction provided for *Lacusovagus* is accurate^45^, this species might indeed be referred to this azhdarchoid clade. This has gained more support with the discovery of four articulated cervical vertebrae that belong to the middle portion of the neck and, as in chaoyangopterids, are elongated, have a low neural spine and lack a lateral pneumatic foramen piecing the centrum^46^. These vertebrae, interpreted to represent cervicals IV to VI, differ from *Meilifeilong youhao* only regarding their proportions since they are of basically the same length, while the cervicals from the Jiufotang Formation show more variation (Fig. 1).

Regarding the limited comparisons that are restricted to the cranial interpretation provided for *Lacusovagus magnificens*^45^, *Meilifeilong youhao* is different by having a wider nasoantorbital fenestra and the presence of a premaxillary sagittal crest.

The last purported species tentatively referred to the Chaoyangopteridae is *Apatorhamphus gyrostega* from the mid-Cretaceous (?Albian-Cenomanian) Kem Kem beds^47^. The specimen consists of a rather fragmentary toothless jaw element interpreted as a probable premaxilla. As known, it is quite hard to work with incomplete specimens and this assignment still needs to be confirmed by better material.

In any case, regarding these occurrences, it should be noted that there are similarities in the pterosaur fauna recorded from the Jiufotang Formation and the Crato Formation with the occurrence of closely related taxa [e.g.,^48,49^], that include tapejarids [e.g.,^31,50^] and anhanguerids^6,51^. The same appears to be happening with the pterosaur fauna preserved in the Kem Kem beds, where tapejarids^52^ and anhanguerids^53^ have also been found. Cretaceous faunal similarities between deposits from Brazil and Africa are somewhat expected, but both sharing several closely related species with Cretaceous deposits in China, to which the Chaoyangopteridae appear to be a recent addition, is quite interesting and needs further investigation.

Lastly, chaoyangopterids are believed to have primarily fed on fishes^54^ and even to have been omnivorous^55,56^, although no direct evidence of their feeding behavior was found so far. In any case, if their suggested feeding strategy is correct, there could have been a dietary niche partitioning among azhdarchoids from the Jehol Biota, since tapejarids are thought to have fed on seeds with their parrot-like beaks^20,57–60^. Further information on this group, as provided by *Meilifeilong youhao*, can shed light on paleoecological aspects, while elucidates the taxonomical diversity of this peculiar group of Jiufotang pterosaurs from China.

## Data Availability

All data generated or analyzed during this study are included in the article.
